# Swedish validation of the Pelvic Floor Questionnaire for pregnant and postpartum women

**DOI:** 10.1007/s00192-022-05264-9

**Published:** 2022-06-29

**Authors:** Ute Jesberg, Annelie Gutke

**Affiliations:** 1 Närhälsan Gibraltar Rehabilitation, Gibraltargatan 1 C, 411 32 Gothenburg, Sweden; 2 Institute of Neuroscience and Physiology, Department of Health and Rehabilitation, University of Gothenburg, 403 50 Gothenburg, Sweden

**Keywords:** Pelvic floor dysfunction, Postpartum, Pregnancy, Questionnaire, Swedish, Validation

## Abstract

**Introduction and hypothesis:**

The German “Pelvic Floor Questionnaire for pregnant and postpartum women” is a self-administered questionnaire customized for pregnancy and the postpartum period that assesses four domains of pelvic floor function regarding perceived symptoms, suffering, and impact on quality of life: bladder, bowel, prolapse, and sexual function. No similar questionnaire is available in Swedish, despite a high prevalence of pregnancy and postpartum pelvic floor dysfunction. Thus, we aimed to translate the validated German questionnaire into Swedish and test its validity and reliability in a Swedish population.

**Methods:**

Translation and cultural adaptation were performed according to guidelines. Of the 248 women who answered the Swedish questionnaire, 57 filled out the questionnaire twice to evaluate test-retest reliability. We also assessed internal consistency and discriminant validity.

**Results:**

The Swedish version of the questionnaire showed good face and content validity. Cronbach’s alpha was in the acceptable to excellent range (bladder 0.82, bowel 0.78, prolapse 0.91, and sexual 0.83), showing adequate internal consistency. A comparison of means (≥ 1 point) showed that the questionnaire significantly (*p* < 0.05) distinguished between women who reported suffering and those who did not. Cohen's kappa for all individual items showed fair to almost perfect agreement (0.24–0.87) between test and retest scores. The intraclass correlation coefficients for domain scores (0.92–0.97) were all in an optimal range.

**Conclusions:**

The Swedish version of the questionnaire is a reliable and valid instrument for assessing pelvic floor disorders, symptom severity, and impact on quality of life during pregnancy and the postpartum period.

## Introduction

Pelvic floor dysfunction is a collective term for several interrelated clinical conditions, including urinary incontinence, fecal incontinence, gas leakage, prolapse, voiding problems, and sexual dysfunction [1]. Pelvic floor dysfunction affects up to 25% of all women [2]. The problems cause suffering for affected women and imply social costs for treatment, aids, and loss of income [3]. Pelvic floor disorders often arise or deteriorate during and after pregnancy [4, 5]. It is important to identify and treat pelvic floor dysfunction early after onset to reduce the risk of long-term problems and aggravating the disorders, as well as to prevent suffering and impaired quality of life [6]. The patient's subjective experience of symptoms needs to be captured because they do not always correlate with the results of anatomical examination [7].

No standardized process is currently available for detecting pelvic floor dysfunction during and after pregnancy. Shame and acceptance often lead to delayed diagnosis and treatment [8]. Due to multifaceted and often interrelated symptoms, history taking and documentation are time consuming for the therapist [7]. Patient-reported outcome measures are important in evaluating pelvic floor dysfunction in women and capturing their experience [8]. Self-administered symptom and condition-specific quality of life questionnaires reflect a patient-centered perspective of symptoms and their severity [9]. In medical research, questionnaires are non-invasive and low-cost, facilitating reproducibility [10]. Questionnaires to evaluate pelvic floor disorders are available, but most of them are limited to one or two specific conditions despite several symptoms usually being inter-related [11].

Only a few questionnaires to evaluate pelvic floor dysfunction have been translated into Swedish. None of these instruments were developed to be applied to pregnant or postpartum women, despite the high prevalence of pregnancy and postpartum pelvic floor dysfunction [2]. The German “Pelvic Floor Questionnaire for pregnant and postpartum women” is a self-administered female pelvic floor questionnaire customized for pregnancy and the postpartum period [12]. It is based on the validated German version of the Australian Pelvic Floor Questionnaire, a complete questionnaire for women of all ages that assesses four domains of pelvic floor function (bladder, bowel, support/prolapse, and sexual function) regarding the perception of symptoms, degree of suffering, and impact on quality of life [1]. The authors of the German questionnaire for pregnant and postpartum women developed additional domains for risk factors during pregnancy and delivery, including questions about the emotional appraisal of birth and postpartum pain. The authors also aimed to adapt the questionnaire to younger women, changing some of the questions and wording from the original questionnaire.

The aims of the present study were to translate the German questionnaire into Swedish and test its validity and reliability in a Swedish population.

## Materials and methods

This study involves two steps: translation and cultural adaptation of the German questionnaire and testing the validity and reliability of the Swedish version. Permission for translation, cultural adaptation, and validation was obtained from the authors of the German questionnaire. This study was approved by the national ethics committee in Uppsala (2020-06-02, ref. no. 2020-01520).

### Translation and cultural adaptation

The translation and cultural adaptation were performed following standardized steps based on guidelines for the cross-cultural adaptation of self-report measures [13]. Two native speakers of Swedish with very good knowledge of German translated the form into Swedish. Both translators were unfamiliar with the original questionnaire, translations were carried out independently, and the translators had no contact with each other before or during the translation. Translator 1 is a mother of two and a physiotherapist with knowledge of the subject. Translator 2 is a 2-month postpartum pharmacist with no specific knowledge of the subject. The two translations were compared with each other, and the translators and author, who is fluent in both languages, agreed on a prefinal version after discussions. Two native speakers of German independently performed back-translations from Swedish to German. Back-translator 1 is a mother of two and a physiotherapist with knowledge of the subject. Back-translator 2 is a mother of three and linguist.

The prefinal version was presented to a committee of experts consisting of three physiotherapists in women's health, a linguist, and an associate professor of physiotherapy specializing in women's health. Translations, back-translations, and the prefinal version were discussed regarding semantics and idiomatic/cultural differences until consensus was reached.

### Validation of the Swedish version

The questionnaire was pilot tested on six patients by cognitive interviewing [14] to ensure valid interpretation and understanding of the questionnaire. Face validity was assured by gradual adaptation according to the patients' suggestions during pre-testing and cognitive interviews. The content validity of the Swedish version was assessed and ensured through discussions and consensus in the committee of experts. The final cross-culturally adapted Swedish version was used for further validation (see Appendix).

Women who were at least 18 years old and between gestational week ≥ 28 to ≤ 12 months postpartum were included in the validation. Exclusion criteria were malignancy or fractures of the pelvic area, urogenital malformations, or medications affecting bladder, miction, or intestinal function.

Both women with and without pelvic floor problems were included in order to measure discriminant validity. Participants were recruited through a social media link with information about the study aim, voluntarism, and anonymity. All women provided consent prior to participation. The link also included contact details in case of questions to the author about the study or participation. A total of 245 women were included in the study.

Internal consistency was measured using Cronbach’s alpha for each domain of the questionnaire. Values of 0.6–0.7 were considered questionable, 0.7–0.8 acceptable, 0.8–0.9 good, and > 0.9 almost excellent [15]. Discriminant validity was assessed using Mann-Whitney-U tests for each individual domain for women with or without subjective suffering. The median scores for each domain were compared for women with or without subjective suffering. The minimum discriminative difference was set to 1 point within a module (sample size *n* ≤ 50) in accordance with the testing of the original questionnaire [12].

Test-retest reliability was determined by Wilcoxon's signed rank test for a subgroup of 57 participants who filled out the questionnaire twice at 1-week intervals. Correlation was tested using Cohen’s kappa for each item on the questionnaire and as the average for each domain. Interpretation of Cohen’s kappa was according to Altman. Any kappa value < 0.2 was considered poor, 0.2–0.4 fair, 0.4–0.6 moderate, 0.6–0.8 good, and > 0.8 very good. The test-retest correlation of the total score for each domain was controlled with the intraclass correlation coefficient (ICC). Values < 0.5 were considered poor reliability, 0.5–0.75 moderate reliability, 0.75–0.9 good reliability, and > 0.90 excellent reliability [17].

## Results

### Translation and cultural adaptation

The process of translation and cultural adaptation implied several minor linguistic changes. The wording of the question about voluntary contraction of the pelvic floor (question 7) in the risk factor module was changed from “consciously” to “willingly” to more clearly indicate the opposite of “reflexively.” The word “prolapse” in the prolapse domain was changed to “heaviness/bulging” because the committee decided that the wording “prolapse” indicates that an actual prolapse has been diagnosed.

According to question 2 in the sexual domain, “If you are not sexually active-why not?” the answer option “partner has problems/impotent” was considered to exclude relationships other than heterosexual and to be outdated. The option was changed to “due to partner” to be more inclusive. It seemed unclear to what kind of sexual activities questions 1, 2, 5, 6, and 7 referred. In the Swedish version, we distinguished between “sex,” meaning all types of sexual activities, and “omslutande samlag” (“embracing intercourse”), a common wording that implies inserting something into the vagina during sexual activity.

Two minor changes were implemented in the answer options in the postpartum part of the questionnaire. Women often do not know their degree of vaginal tearing, so the option to answer “don’t know” was added in question 6. An option of “not applicable” was added to question 9 because it was assumed that not all women feel fear during childbirth.

According to the pre-test, most of the women had difficulties understanding “contract the pelvic floor” and the word “knipa” (“pinch”), which is common in Swedish colloquial language, was added. In the domain heaviness/bulging, several women commented on the wording of question 1, “something unfamiliar is bulging in the vagina,” and they also wished for a question about chafing. The question was changed to “Do you have the sensation of bulging or chafing?”

Out of six women, four commented on the answer options for question 1 in the sexual domain. They felt that the option “sometimes” was missing for the question about vaginal lubrication. To avoid changes in the scoring system, “usually” was added to the wording of the question.

Regarding question 2 in the sexual domain, a question about vaginal sensation during intercourse, women commented that option 1, “feel a lot,” can also mean “feel a lot of pain,” which corresponds to option 4, “feel pain.” The wording was changed to “normal/pleasant,” which also matches the wording in the Australian Pelvic Floor Questionnaire [9].

### Validation

**Fig. 1:**
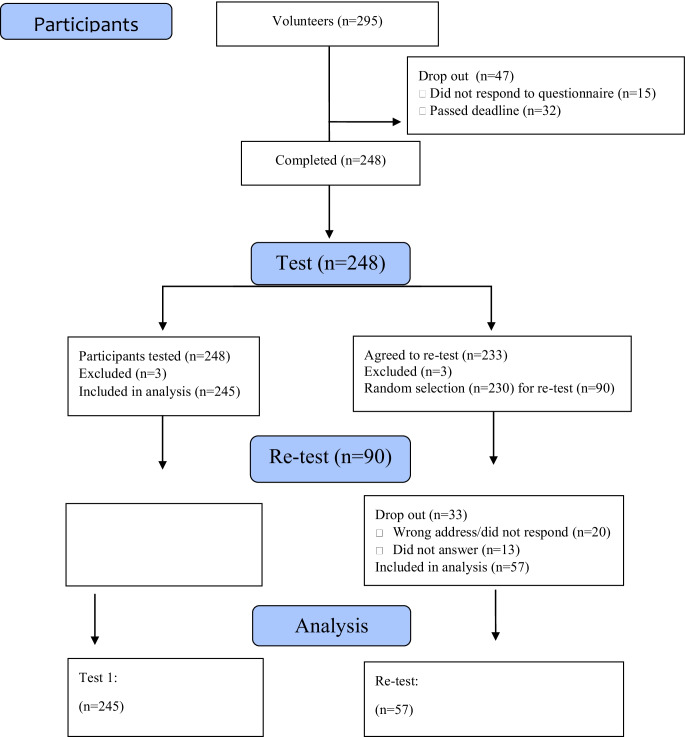
Recruitment process and study participants

The validation process included 245 women, 57 of whom were included in the assessment of test-retest reliability (Fig. 1).

Demographics were comparable between the test and retest-groups (Table 1).

**Table 1:** Characteristics of the test and re-test groups

	Test n=245	Re-test *n* = 57
Age, years	31.8 (20-41)	31.3 (26-40)
Status		
Gestational week		
• 28-33	30 (12.2)	4 (7)
• 34-39	19 (7.8)	6 (10.6)
• 40-41	4 (1.6)	3 (5.3)
Months postpartum		
• 0-2	42 (17.1)	14 (24.6)
• 3-5	54 (22.0)	11 (19.3)
• 6-8	50 (20.4)	12 (21.0)
• 9-12	46 (18.8)	7 (12.3)
Number of children		
• 1	128 (46.7)	31 (54.4)
• 2	73 (26.6)	17 (29.8)
• 3	19 (16.9)	5 (8.8)
• 4 or more	3 (1.1)	1 (1.8)
Mode of birth		
• Ventouse/forceps	27 (9.9)	5 (8.8)
• Cesarean section (1 or more)	47 (17.2)	9 (15.8)
• Vaginal tear, degree 3 or 4	21 (7.7)	3 (5.3)
Geographic area		
• City	99 (40.4)	18 (31.6)
• Suburbs/outskirts	40 (16.3)	8 (14.0)
• Smaller city	58 (23.7)	16 (28.0)
• Small village	26 (10.6)	9 (15.8)
• Countryside	22 (9.0)	6 (10.5)
Education		
• Elementary school	2 (0.8)	0 (0)
• Secondary education	26 (10.6)	7 (12.3)
• College/university ≤ 3 years	17 (6.9)	4 (7.0)
• College/university ≥ 3 years	200 (81.6)	46 (80.7)

Values are *n* (%) or mean (range)

Cronbach’s alpha values showed acceptable agreement for bowel symptoms, good agreement for bladder and sexual symptoms, and very good internal consistency for pelvic organ prolapse [15] (Table 2).

**Table 2:** Internal consistency and discriminant validity for each domain

Domain	Internal consistency	Discriminant validity	
	Cronbach’s alpha	Suffering	Median score
Bladder	0.82	Yes	2.08
		No	0.62
Bowel	0.78	Yes	2.58
		No	1.61
Prolapse	0.91	Yes	2.67
		No	1.33
Sexual symptoms	0.80	Yes	2.50
		No	1.25

The questionnaire was able to distinguish (*P* < 0.05) between women who reported suffering and those who did not. For women who reported little to much suffering in the domains of bladder, pelvic organ prolapse, and sexual symptoms, the median score was at least 1 point higher than in women who did not report suffering, which corresponds to the minimal important difference established in the validation of the German questionnaire [12]. In the bowel domain, the median was 0.97 points higher in women with subjective suffering (Table 2).

Wilcoxon' s signed rank test for each domain confirmed the null hypothesis (Table 3), and the comparison of median scores at test and retest showed no significant differences (Table 2). This indicates good test-retest reliability of the questionnaire.

**Table 3:** Test-retest reliability based on Wilcoxon’s signed rank test, Cohen’s kappa, and intraclass correlation coefficient (ICC)

Domain	*P*-value	Kappa (item range) Mean (range)	ICC (95% CI) (score sum) Mean (range)
Bladder	0.753	0.58 (0.24-0.73)	0.948 (0.911-0.969)
Bowel	0.135	0.64 (0.41-0.87)	0.948 (0.911-0.969)
Pelvic organ prolapse	0.911	0.49 (0.45-0.60)	0.918 (0.860-0.952)
Sexual symptoms	0.529	0.69 (0.48-0.78)	0.973 (0.953-0.984)

Test-retest reliability was further investigated using Cohen’s kappa (Table 3) for each individual item in the questionnaire. The kappa values showed moderate to very good agreement, with one exception [16]. The exception was the question of how much the bladder symptoms affect or limit daily life. The kappa value of 0.24 indicated only fair agreement between the test and re-test for that individual item. Items in the bowel domain showed moderate to almost perfect agreement, whereas items in the pelvic organ prolapse domain agreed moderately and all items in the domain for sexual symptoms showed moderate to substantial agreement between the test and retest.

We found no significant difference between test and retest. The test-retest comparison of the summed scoring for each domain using the ICC indicated excellent correlation [17] (Table 3).

## Discussion

The Swedish version of the questionnaire showed good face validity and content validity. The statistical evaluation showed good internal consistency, discriminant validity, and test-retest reliability.

### Translation and cultural adaptation

In the translation process, some difficulties arose regarding the sexual domain, as the approach and wording in the original questionnaire were interpreted as being somewhat outdated and non-inclusive. The criticism of the questionnaire that emerged from both translators, the expert committee, and pre-test participants was that the wording presumes a male partner and that sex includes penetration. The sexual domain also turned out to be the domain most discussed and commented on during the pilot tests. A large number of study participants added additional information about reasons for not being sexually active. This indicates that it may be necessary to give the patient the opportunity to provide explanations and clarifications in the clinic in order to get a clearer picture of sexual problems. It also clearly shows that the sexual topics often lack distinct formulations and that the wordings that exist can be interpreted very differently by different people.

Concerning the postpartum module, several participants commented on the fact that the questions presumed that all women experience fear and pain during labor, but that the questionnaire did not take fear before giving birth into account. This may be due to cultural differences between Sweden and Germany.

The phenonemon of fear of childbirth is a current subject discussed in research and media in Sweden. A recent systematic review [18] showed that Sweden has had more research about fear of childbirth in the last 2 decades than any other country. This does not necessarily imply that Swedish women have more fear of giving birth, but it shows that Swedish researchers and clinicians in the field consider fear of childbirth an important issue to be investigated and addressed. According to several studies analyzed in the review, Sweden showed higher prevalence of fear of childbirth compared to other European countries. However, the prevalence of fear of childbirth in Germany is not known because there are no studies about fear of childbirth in Germany available yet. Obstetric injuries and insufficient maternal care have been frequently discussed in Swedish media in recent years, and negative impressions of childbirth and maternal care can be one important factor in generating fear of childbirth [18–20]. These factors might explain why Swedish women in this study consider fear of childbirth to be an important aspect to take into account when inquiring perceived fear related to giving birth.

In addition, the participants wished to be able to report birth-related complications other than perineal tears, which suggests that the grade of perineal tearing is not the only factor impacting birth experience and birth-related problems for women. Studies have confirmed that higher grade of perineal tear increases the risk of postpartum pelvic floor dysfunction, but adverse functional effects are experienced by women with perineal lacerations of all grades, as well as by those with an intact perineum [21]. Second-degree trauma has been shown to be a risk factor for urinary incontinence, whereas inflammatory states and infections can be possible causes of dyspareunia [21]. Therefore, it is important to take other types of complications into account, such as infections of the urinary tract or uterus, pain in the pelvis and the tailbone, hemorrhoids, and nerve injuries.

### Validation

The results of the validation of the Swedish questionnaire are comparable to the results of the validation of the German questionnaire [13]. Internal consistency was at least acceptable in the Swedish version for all domains: bladder (original: 0.775 vs. Swedish 0.821), bowel (0.695 vs. 0.783), prolapse (0.745 vs. 0.913), and sexual function (0.63 vs. 0.809). In addition, the discriminant validity for the domains was comparable for both versions according to the comparison of median test and retest scores.

Correlations of the domains were between good and excellent in both the Swedish and German versions according to ICC values: bladder (original: 0.818 vs. Swedish: 0.948), bowel (0.874 vs. 0.948), prolapse (0.801 vs. 0.918), and sexual function (0.732 vs. 0.973). One exception was the sexual function domain of the German version, which was classified as moderately reliable. An explanation for the higher values for the Swedish version can be that some changes were implemented in the sexual domain during the translation and cultural adaptation process because of comments from pre-testing patients and discussions by the committee of experts. These changes may have made the Swedish version more intelligible and more relatable for women, regardless of sexual orientation.

The evaluation of the test-retest reliability for the bladder domain showed only fair agreement between for the question about limitations in daily life related to bladder symptoms (0.24). This is a considerably lower value than for the other questions, and what leads to this discrepancy is unclear. The comparison of test and retest values showed that < 1% (*n* = 5) of the participants reported increased limitations after 1 week, whereas 30% (*n* = 17) reported a reduction of limitations. The test-retest reliability according to kappa values was slightly lower for the Swedish version than the German version. This could be due to different intervals between test and retest for the two studies. In the German study, the interval was 1 day, whereas a 1-week interval was used in the Swedish study. The rapid physical changes that occur during pregnancy and the postpartum period may have affected the agreement between reported symptoms at the two time points.

The original German questionnaire has recently been translated and validated in Turkish [22] and Italian [23], which corroborates the need for patient-reported outcome measures of pelvic floor dysfunction specifically designed for pregnancy and the postpartum period. The recent translations and validations have shown similarly positive statistical results.

A limitation of this study is that the majority of the participants came from the metropolitan area and had a university degree. This may have affected their understanding of the questions and the wording and expressions. A broader target group with a more varied background and more basic knowledge of the Swedish language would be desirable. Despite advertising via a national blog and recruitment at a rehabilitation center in a socially vulnerable and multicultural area, the study sample turned out to be a relatively homogeneous group in terms of educational background.

Another limitation may be that the participants were recruited via social media, mainly on websites that focus on information about women’s health. This implies a risk that the recruited women had an interest in and prior knowledge of the pelvic floor and pelvic floor dysfunction. However, information about the study was also distributed via private links and accounts to reach a more mixed target group, and a large proportion of participants had no pelvic floor issues. This may have affected the results of the test-retest validity.

One strength of this study is the relatively large number of participants in the test and retest groups. There is also a good spread among the participants regarding age, gestational week, postpartum months, number of children, and mode of delivery.

A valid and reliable self-administered Swedish questionnaire to assess all areas of pelvic floor dysfunction in pregnant and postpartum women is required and requested by therapists in Sweden. Hopefully, it will fill a gap in the clinic and research and facilitate history taking and the documentation of symptoms and the evaluation of treatment effects for physiotherapists.

In summary, the Swedish version of the German “Pelvic Floor Questionnaire for pregnant and postpartum women” was found to have face validity and content validity, as it is culturally and linguistically understandable by Swedish pregnant and postpartum women. The questionnaire has good internal consistency and test-retest reliability regarding both individual questions and score sums for the separate domains. The questionnaire discriminantly distinguishes women with or without subjective suffering. Thus, the Swedish version can be recommended for use in clinics and research.

U. Jesberg: Project development, data collection, data analysis, manuscript writing

A. Gutke: Project development, data analysis, manuscript writing

## Appendix

Bäckenbotten-frågeformulär för gravida och kvinnor efter barnafödande
